# Towards a single-assay approach: a combined DNA/RNA sequencing panel eliminates diagnostic redundancy and detects clinically-relevant fusions in neuropathology

**DOI:** 10.1186/s40478-022-01466-w

**Published:** 2022-11-17

**Authors:** Cheyanne C. Slocum, Hyeon Jin Park, Inji Baek, Jeff Catalano, Martin T. Wells, Benjamin Liechty, Susan Mathew, Wei Song, James P. Solomon, David J. Pisapia

**Affiliations:** 1 Weill Cornell Medical College, New York, NY USA; 2 Department of Pathology and Laboratory Medicine, Weill Cornell Medicine, 1300 York Avenue, New York, NY 10065 USA; 3 Department of Statistics and Data Science, Cornell University, Ithaca, NY USA

**Keywords:** CNS neoplasms, Next-generation sequencing, Molecular diagnostics

## Abstract

Since the introduction of integrated histological and molecular diagnoses by the 2016 World Health Organization (WHO) Classification of Tumors of the Nervous System, an increasing number of molecular markers have been found to have prognostic significance in infiltrating gliomas, many of which have now become incorporated as diagnostic criteria in the 2021 WHO Classification. This has increased the applicability of targeted-next generation sequencing in the diagnostic work-up of neuropathology specimens and in addition, raises the question of whether targeted sequencing can, in practice, reliably replace older, more traditional diagnostic methods such as immunohistochemistry and fluorescence *in-situ* hybridization. Here, we demonstrate that the Oncomine Cancer Gene Mutation Panel v2 assay targeted-next generation sequencing panel for solid tumors is not only superior to IHC in detecting mutation in IDH1/2 and TP53 but can also predict 1p/19q co-deletion with high sensitivity and specificity relative to fluorescence *in-situ* hybridization by looking at average copy number of genes sequenced on 1p, 1q, 19p, and 19q. Along with detecting the same molecular data obtained from older methods, targeted-next generation sequencing with an RNA sequencing component provides additional information regarding the presence of RNA based alterations that have diagnostic significance and possible therapeutic implications. From this work, we advocate for expanded use of targeted-next generation sequencing over more traditional methods for the detection of important molecular alterations as a part of the standard diagnostic work up for CNS neoplasms.

**Supplementary Information:**

The online version contains supplementary material available at 10.1186/s40478-022-01466-w.

## Introduction

Beginning with the 2016 World Health Organization (WHO) Classification of Tumors of the Central Nervous System (CNS), there was a formalized expansion of the use of integrated histologic and molecular diagnoses in neuropathology [29, 31, 36]. For example, oligodendrogliomas are defined by the presence of both isocitrate dehydrogenase (IDH) mutation (either *IDH1* or *IDH2*) and 1p/19q co-deletion irrespective of the morphology of an infiltrating glioma, while adult infiltrating gliomas without 1p/19q co-deletion are separated into IDH-mutant and IDH-wildtype subgroups, the latter in the adult setting now exclusively termed “glioblastoma” [7, 10, 15, 22, 28, 31, 32, 36, 39, 45, 48].

When it was established that co-deletion of chromosomal arms 1p/19q molecularly distinguished oligodendroglioma from IDH-mutant astrocytoma, fluorescence in situ hybridization (FISH) became a staple in the diagnostic work-up of infiltrating gliomas [10, 22, 28]. The utility of FISH in the work-up of infiltrating gliomas expanded with the recognition of additional copy number and structural variants that have prognostic significance and therefore can confer designation of a higher overall grade independent of histological features. As initially detailed in the cIMPACT-NOW update 3 [9] along with *TERT*-promoter mutation, the presence of *EGFR* amplification, and/or the combination of chromosome 7 gain with chromosome 10 loss allows for a grade 4 designation in IDH-wildtype infiltrating astrocytoma irrespective of the presence of necrosis or microvascular proliferation [1, 3, 7, 9, 10, 22, 32, 41, 46]. Similarly, homozygous deletion of *CDKN2A* in IDH-mutant infiltrating astrocytoma confers a grade 4 diagnosis even in the absence of other high grade histological features, as described in the cIMPACT-NOW update 5 [3, 4, 7, 8, 14, 32, 38]. Additionally, there is evidence that homozygous loss of *CDKN2A* may also be a negative prognostic indicator in oligodendrogliomas [4].

Due to its close correlation with clinical outcomes, IDH mutational status has transitioned from a prognostic role to become incorporated as a gold-standard diagnostic marker. IDH mutation is found by definition in all oligodendrogliomas and in 60–80% of infiltrating astrocytomas with lower grade histological features [10–12, 15, 22, 26, 31, 37, 39, 48]. Grade 4 astrocytomas with IDH mutation (previously termed ‘Glioblastoma, IDH-mutant’) tend to occur in younger patients with a mean age of 32 years at diagnosis and confer a median overall survival of 31 months compared to a mean age of diagnosis at 59 years and median overall survival of 15 months for IDH-wildtype glioblastoma [10, 11, 15, 22, 26, 31, 36, 37, 39, 45, 48]. Mutation can occur in the genes coding for either *IDH1* or *IDH2* genes, but in over 90% of infiltrating gliomas with IDH mutation, the detected variant is *IDH1* R132H [7, 11, 12, 25, 26, 36, 39, 48]. Given the high frequency of the *IDH1* R132H variant over others, immunohistochemical stains targeting this specific alteration have been readily available since 2010 [11, 12, 15, 25, 26, 37]. In the remaining 10% of IDH-mutated cases, the most common IDH variants include *IDH1* R132C/S/V/G and *IDH2* R172H, with mutations in *IDH2* occurring more frequently in oligodendrogliomas [7, 11, 15, 25, 26, 48]. Because IDH-mutant infiltrating astrocytoma also commonly harbor *TP53* and *ATRX* mutations, these genes can act as additional markers for distinguishing between IDH-mutated astrocytomas and oligodendrogliomas. Finally, loss of *ATRX* is typically mutually exclusive with *TERT*-promoter mutations, the latter of which are commonly found in both oligodendrogliomas and glioblastoma, and *TP53* mutations are only very rarely found in cases of oligodendroglioma [10, 15, 22, 26, 48].

Particularly at academic centers in developed nations, the use of next-generation sequencing (NGS) has become increasingly common in pathology laboratories as improvements in technology have decreased cost, allowing for easier adoption and integration into clinical practice [29, 47]. Targeted NGS allows for the concurrent sequencing of panels of genes [29, 47] and many different targeted panels are now available commercially, with some designed to cover genes commonly altered over a diversity of solid and/or hematopoietic tumor types. The Oncomine Cancer Gene Mutation Panel v2 assay is a targeted NGS panel that assesses over 2,500 amplicons from 143 genes commonly mutated in solid tumors. In addition to the detection of single nucleotide variants, insertions, deletions, and copy number alterations from DNA, the assay also includes an RNA sequencing component for the detection of selected fusion transcripts for which both gene partners and their common breakpoints are known a priori.

Despite the increased use of targeted NGS in pathology, more traditional methodologies such as immunohistochemistry (IHC) and FISH are still often used concurrently during standard work-up of pathology specimens. The use of multiple diagnostic modalities may result in the generation of redundant information with little clinical utility. In other instances, there may be disparate results between two different modalities creating diagnostic uncertainty and prompting the question of which assay should be regarded as the gold standard. The logistic and diagnostic challenges posed by using multiple different molecular assays poses the question as to whether diagnostic neuropathology can be streamlined by replacing, rather than complimenting, traditional techniques with targeted NGS. Several groups have reported on different approaches for using targeted-NGS to replace FISH for the detection of 1p/19q co-deletion [18, 21, 34] and others have assessed if targeted-NGS could alone be used to diagnose infiltrating gliomas [29].

In this study, we sought to determine the diagnostic utility of targeted-NGS using the Oncomine Comprehensive Panel v2 (referred to hereafter as Oncomine) in the setting of a single institution large academic center’s neuro-oncology practice, and in particular if the panel obviates the need for IHC and FISH, without loss of clinically important information. We assess the ability of Oncomine to detect mutations in IDH and TP53 relative to IHC, and to predict ATRX status. Additionally, we compare copy number data obtained from FISH with that derived from targeted NGS for selected loci including *EGFR* and *CDKN2A* as well as genes located on chromosomes 1 and 19. Finally, we examined the extent to which the RNA component of the Oncomine panel adds value to the work-up of infiltrating gliomas and to CNS tumor entities more broadly.

## Methods and materials

### Cohort selection

The study cohort comprises 233 neurosurgical cases over 231 patients resected at New York-Presbyterian Hospital/Weill Cornell Medicine. The cases included represent all cases for which the Oncomine panel was performed over a 19-month period irrespective of age or diagnosis. The number of cases in each diagnostic class as well as the sex and age distribution of infiltrating glioma cases are listed in Table 1. Cases with no mutations detected on either the DNA and RNA portion of the Oncomine panel are noted in Table 2 and represent 11.2% (26/233) of all cases submitted for sequencing. Chart review and data collection were conducted with approval by the Institutional Review Board at Weill Cornell Medicine (IRB #: 1,312,014,589).

**Table 1:** Diagnoses of cohort patients along with sex distribution for cases of infiltrating glioma and average age of diagnosis for infiltrating gliomas

Total number of patients for all tumor classes	
Diffuse astrocytoma, IDH-wildtype, presenting in adult patients	112
Glioblastoma, IDH-wildtype (grade 4 histology)	99
Glioblastoma, IDH-wildtype (lower grade histology)	9
(Pediatric-type) Diffuse hemispheric glioma, H3G34-mutant	3
Diffuse astrocytoma, IDH-wildtype, NOS	1
Astrocytoma, IDH-mutant	29
Neuroepithelial Tumor, NOS	16
Oligodendroglioma	14
Pilocytic Astrocytoma	11
Diffuse gliomas, presenting in pediatric patients	6
Diffuse midline glioma, H3K27M mutant	4
Diffuse hemispheric glioma, H3G34-mutant (pediatric patients)	2
Astrocytoma, NOS	6
Meningioma	5
Ganglioglioma	4
Dysembryoplastic Neuroepithelial Tumor (DNET)	3
Ependymoma	3
Medulloblastoma	2
Non-Diagnostic	2
Solitary Fibrous Tumor/Hemangiopericytoma (SFT/HPC)	2
Central Neurocytoma	1
Cortical Dysplasia	1
Craniopharyngioma	1
Diffuse Leptomeningeal Glioneuronal Tumor (DLMGNT)	1
Dysplastic Cerebellar Gangliocytoma	1
Embryonal Tumor with Multilayered-Rosettes, C19MC-altered	1
(ETMR, C19MC-altered)	1
Endodermal Cyst	1
Epilepsy	1
Malignant Neoplasm, NOS	1
Meningioangiomatosis	1
Pilomyxoid Astrocytoma	1
Pineal Parenchymal Tumor of Intermediate Differentiation (PPTID)	1
Pituicytoma	1
Reactive	1
Schwannoma	1
Tuber	1
*Sex of infiltrating glioma cases in adults*	
Oligodendroglioma	
M	10
F	4
IDH-mutated Astrocytoma	
M	20
F	9
Diffuse astrocytomas, IDH-wildtype	
M	51
F	61
*Average age at diagnosis for infiltrating glioma cases in adults*	
Oligodendroglioma	48.5 ± 13.5
Astrocytoma, IDH-mutant	38.2 ± 12.1
Diffuse astrocytomas, IDH-wildtype	61.9 ± 13.0

**Table 2:** Distribution of cases for which *no variants* (DNA or RNA) were detected on the Oncomine panel by tumor class

Total number of cases for per tumor class	
Neuroepithelial Tumor, NOS	5
Astrocytoma, NOS	3
Dysembryoplastic Neuroepithelial Tumor (DNET)	2
Ependymoma	2
Non-Diagnostic	2
Cortical Dysplasia	1
Endodermal Cyst	1
Epilepsy	1
Embryonal Tumor with Multilayered Rosettes C19MC-altered (ETMR, C19MC-altered)	1
Ganglioglioma	1
Diffuse astrocytoma, IDH-wildtype, NOS	1
Meningioma	1
Pilocytic Astrocytoma	1
Pilomyxoid Astrocytoma	1
Pineal Parenchymal Tumor with Intermediate Differentiation (PPTID)	1
Reactive	1
Schwannoma	1
Solitary Fibrous Tumor/Hemangiopericytoma (SFT/HPC)	1

### IHC methods

The clone, dilution, and antigen retrieval for the IDH1 R132H, p53, and ATRX immunohistochemical stains are shown in Additional file 1: Table S1.

### Scoring of p53 immunohistochemical staining

Pathology reports were reviewed for cases in which p53 immunostaining was conducted. Staining analysis was conducted by a board-certified neuropathologist with subspecialty training prior to the availability of the Oncomine sequencing results. A score of 0 was assigned to cases wherein tumor cells were completely negative for p53 labeling, suggestive of a truncating mutation; a score of 1 was assigned for a pattern of labeling characteristic of tumors with wildtype TP53 (i.e. weak labeling in scattered cells), a score of 2 was assigned to cases wherein labeling was deemed ambiguous and confirmatory sequencing was recommended, and a score of 3 was assigned to cases exhibiting strong labeling in the majority of tumor cells, suggestive of an underlying missense mutation. Examples of the staining pattern for each score can be seen in Additional file 2: Fig. S1.

### FISH methods

FISH was performed on paraffin section slides using the locus specific probes TP73 (1p36.32), ANGPTL1 (1q25.2), ZNF443 (19p13.2) and GLTSCR1 (19q13.33) (Vysis/Abbott Molecular Inc., Des Plaines, IL) to rule out co-deletion of TP73 (1p36.32) and GLTSCR1 (19q13.33) genes. FISH was also performed using the EGFR (7p11.2) and CEP 7 (p11.1-q11.1) probes (Vysis/Abbott Molecular Inc., Des Plaines, IL) to rule out an amplification of EGFR gene. Hybridization was performed according to the manufacturer’s specifications. Two hundred interphase nuclei were screened for each probe.

### Oncomine methods

Total DNA was extracted from up to ten pooled paraffin sections using Maxwell® 16 DNA purification kits according to the manufacturer’s instructions (Promega, Madison, WI). Total RNA was extracted from up to five pooled paraffin sections using Qiagen RNeasy FFPE kit (Hilden, Germany) according to the manufacturer’s instructions with modification of deparaffinization steps to use Hemo-De (Electron Microscopy Sciences, Hatfield, PA). Complementary DNA (cDNA) was synthesized from the extracted RNA using the SuperScriptä VILOä cDNA synthesis Kit (Thermo Fisher Scientific, Waltham, MA). Amplicon libraries were prepared manually using the Ion AmpliSeq™ Library Kit 2.0 following manufacturer’s instructions (Thermo Fisher Scientific). The amplicons were ligated to Ion Xpress™ Barcode Adapters (Thermo Fisher Scientific) and purified using AMPure XP beads (Beckman Coulter, Brea, CA). Quantification of the purified libraries was performed with the Ion Library TaqMan™ Quantitation Kit (Thermo Fisher Scientific), and sequencing libraries were pooled at a concentration of 50 pM. Sequencing runs included a maximum of 18 DNA and RNA samples in addition to two positive controls, HD200 and HD796 (Horizon Dx). Sequencing was performed on an Ion 540™ chip using the Ion Chef™ System and the Ion 540™ Kit-Chef on the Ion S5™ XL Sequencing Systems (Thermo Fisher Scientific). Sequencing data were analyzed on a Torrent Server through the Torrent Suite Software. Reads were aligned to the hg19 human reference genome and variants were analyzed with the Ion Reporter Software (Thermo Fisher Scientific). A list of genes covered by this panel are included in Additional file 1: Table S2.

### Determination of copy number and statistics

The raw copy number for genes sequenced by the Oncomine panel located on chromosomes 1p, 1q, 19p, and 19q were averaged for each case of infiltrating glioma. ANOVA and unpaired 2 tailed t-tests were performed using Microsoft excel software to determine statistical significance between groups (i.e. oligodendroglioma, IDH-mutated infiltrating astrocytoma, and IDH-wild type infiltrating astrocytoma) for average copy number of genes sequenced on each arm of chromosomes 1 and 19. Significance was defined as a *p*-value < 0.05.

The receiver operating characteristic (ROC) curve for comparing copy number assessment of 1p and 19q from NGS relative to FISH was generated using Stata software. The ROC curve was used to determine threshold values for average copy number with the highest sensitivity and specificity for determining 1p/19q co-deletion status relative to FISH.

## Results

### Cohort demographics

The case cohort comprised 233 surgical resections from 231 patients for which the Oncomine panel was completed. Of the 231 patients, 122 were male and 109 were female, and 27 cases were from pediatric patients (age at diagnosis < 21 years). Of 157 cases diagnosed as infiltrating gliomas presenting in adult patients, 14 were oligodendroglioma, 29 were IDH-mutated infiltrating astrocytoma, and 112 were IDH-wildtype infiltrating astrocytomas. Within the latter category, these 112 cases included the following subsets: 99 cases of glioblastoma, IDH-wildtype with histological features of GBM; 9 cases with lower-grade histological features but meeting molecular criteria for GBM, IDH-wildtype; 3 cases of pediatric-type diffuse hemispheric glioma, H3G34-mutant, and 1 case of IDH-wildtype astrocytoma with grade 3 histological features, NOS (i.e. not meeting molecular criteria for GBM). Tremaining 76 cases comprisedentities other than infiltrating gliomas arising in adults (Table 1).

### Detection of IDH and TP53 by immunohistochemistry compared to oncomine

#### IDH

**Fig. 1:**
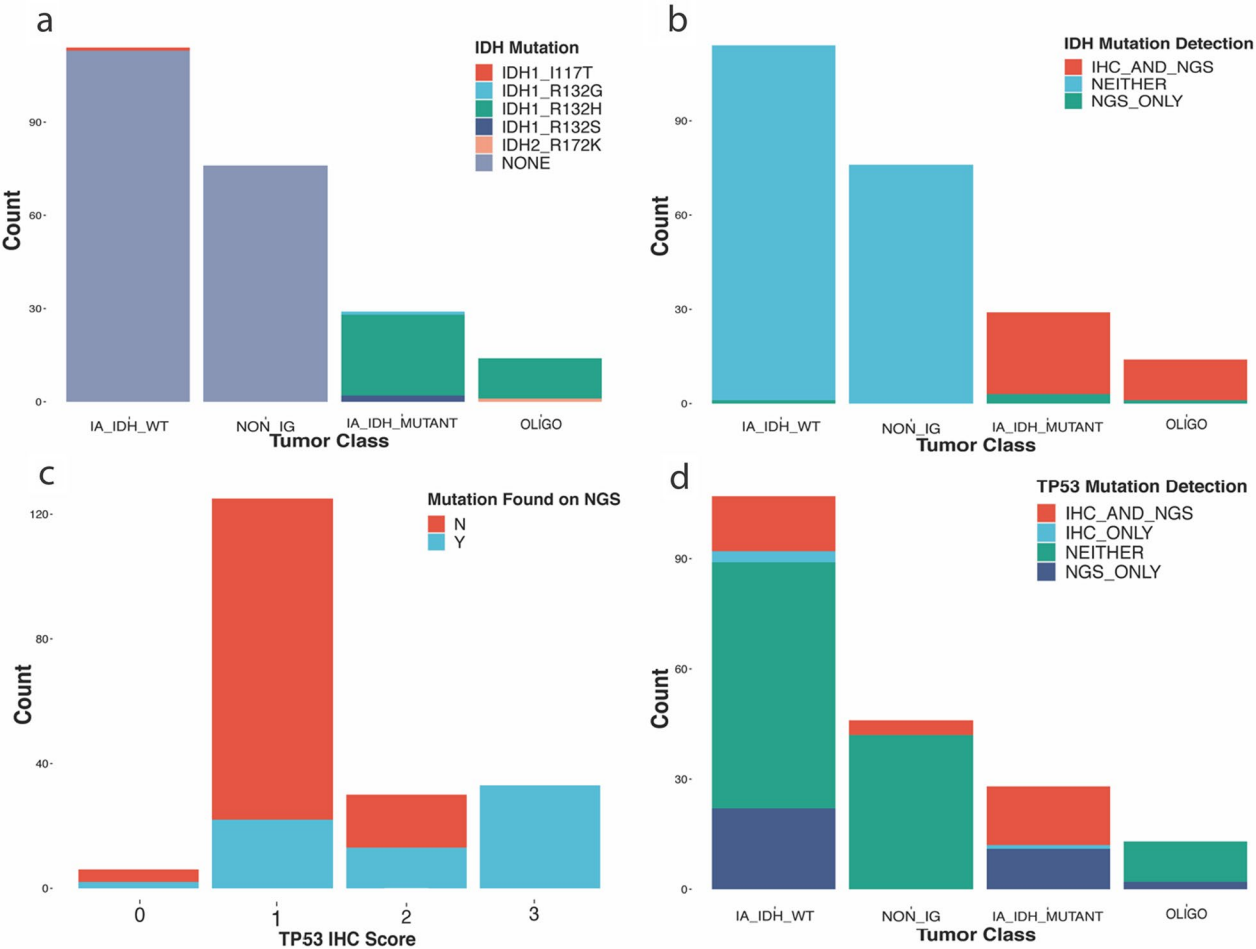
Comparison of IDH and TP53 mutations detected by immunohistochemistry (IHC) and targeted next-generation sequencing (NGS) using the Oncomine Panel. **A** Detection of IDH mutation in infiltrating gliomas by IHC alone, NGS alone, or both, (**B**) codon change of IDH mutations detected by the Oncomine targeted NGS panel, (**C**) concordance of TP53 IHC score with TP53 mutation detection on NGS, (**D**) detection of TP53 mutation by IHC, NGS, both, or neither by class of infiltrating glioma and all remaining diagnoses. (IA_IDH_WT = IDH-wildtype infiltrating astrocytoma in adults; IA_IDH_MUTANT = IDH-mutant infiltrating astrocytoma; OLIGO = oligodendroglioma; NON_IG = non-infiltrating glioma)

Immunohistochemistry for IDH1 R132H IHC stain was performed on 191 of the 233 cohort cases. Of these 191 cases, 152 were negative for IDH1 R132H mutation and 39 were positive. All cases positive for IDH R132H by IHC also had this mutation detected by Oncomine while 5/152 cases which were negative for the R132H mutation by IHC had an alternative IDH1/2 mutation detected by the Oncomine panel (Fig. 1a, b). Of the additional 5 IDH mutations detected by Oncomine but not IHC, none were IDH1 R132H. These included an IDH2 R172K alteration detected in an oligodendroglioma, as well as IDH1 R132G (1 case) and IDH1 R132S (2 cases) mutations, all three of which were seen in IDH-mutant infiltrating astrocytomas (Fig. 1a, b). Finally, a single case revealed an IDH1 I117T alteration by Oncomine. Since this alteration does not have a known pathogenic association and other molecular alterations in this tumor were characteristic of an IDH-wildtype infiltrating astrocytoma, the mutation was regarded as clinically insignificant and not diagnostic of the IDH-mutated class of tumors.

Altogether these results yield a 90.7% sensitivity and 100% specificity for IDH1-R132H immunohistochemical staining relative to the detection of IDH1/IDH2 alterations overall as determined by Oncomine sequencing. Importantly, our data in this series is consistent with a sensitivity and specificity of 100% for the Oncomine panel in detecting pathogenic IDH alterations. No case that was negative for IDH alterations by Oncomine showed positive IDH staining, and moreover none of these cases demonstrated evidence of IDH alterations by concurrent NGS testing by alternative platforms (e.g. Foundation Medicine in a subset of cases) or displayed clinical, histological or other molecular features that would raise suspicion of a false-negative sequencing result (data not shown). In this cohort 9.3% of the total pathogenic IDH1/2 mutations (4/43) were missed by IHC, as expected given its specificity for IDH1 R132H.

#### TP53

IHC staining for p53 was conducted for 194 of the 233 cases. A score of 0–3 was assigned to each case (as described in Methods). Of the 194 p53 IHC stains conducted, the distribution of scores is shown in Fig. 1c, along with the associated TP53 alterations called by Oncomine.

Two of six (33%) cases that were given a score of 0, indicating that a truncating mutation was considered, had TP53 mutations detected by Oncomine. One case indeed harbored a frameshift truncating mutation and the other case harbored 2 distinct missense mutations. Of the cases given a score of 1, 22/125 (18%) had at least one TP53 alteration detected and 5 cases had more than 1 TP53 mutation; in total there were 17 missense mutations, 6 frameshift deletions, 4 splice site alterations, and 2 nonsense mutations. Out of the 30 cases given a p53 staining score of 2, 13 cases had TP53 mutations all of which were missense, 4 of these cases had 2 missense mutated detected. All of the cases scored 3 (33/33; 100%) had either a missense mutation detected (32 cases) or a non-frameshift deletion (N131del; 1 case). Five of these cases had compound heterozygous mutations, with concurrent nonsense seen in 4 cases and a frameshift mutation detected in 1 case.

TP53 mutation was suspected if the IHC was scored as 0 (completely absent staining suggestive of a truncating mutation) or 3 (strong labeling consistent with a missense mutation). When cases with a score of 0 or 3 are considered in aggregate, 35/39 cases ultimately did have TP53 mutations detected by the Oncomine panel, resulting in 89.7% positive predictive value for mutation by IHC. A score of 1 was given if the pattern of labeling was considered most consistent with wildtype TP53 mutation. 103/125 indeed lacked a TP53 mutation as detected by Oncomine, yielding a positive predictive value of 82% for wildtype TP53 given a score of 1. The cases scored as 1 that did ultimately reveal TP53 mutations often harbored either non-missense mutations or harbored compound heterozygous mutations as stated above, potentially accounting for the discrepancy in a majority of these cases. Given that a score of 2 represents an ambiguous staining pattern, as expected roughly half of the cases with a score of 2 (13/30 or 43%) harbored a TP53 mutation and the rest did not. In summary, if the number of cases scored as 2 (ambiguous staining) are added to those with scores 0,1 and 3 that showed discrepant sequencing results, we obtain a total of 56/194 (29%) cases for which p53 IHC failed to predict the sequencing result. The stain was most reliable when strong labeling was present in a majority of tumor cells, suggesting a missense mutation, and was considerably less reliable in predicting TP53 mutations in the context of the other staining patterns.

### Prediction of ATRX mutational status based on IDH and TP53 mutations detected by oncomine

The Oncomine panel does not directly assess for alterations in *ATRX*, the loss of which is closely associated with the presence of concurrent *IDH* and *TP53* mutations in the majority of infiltrating gliomas arising in adults. It has been reported that IDH-mutant astrocytomas have co-occurring *TP53* mutations in 94% of cases and loss of ATRX expression in 86% of cases [10].We sought to assess the added value of performing ATRX immunohistochemistry over and above an *ATRX* prediction metric based upon the status of *IDH* and *TP53* alterations as determined by Oncomine alone in the adult population within our cohort. The ATRX prediction status was compared to results of ATRX IHC as well as Foundation Medicine targeted next-generation sequencing panel results (which directly assesses ATRX) when available (78 cases).

Of the 29 IDH-mutant gliomas without 1p/19q codeletion 28/29 had a TP53 mutation detected. It was predicted that all 28 of the IDH-mutant infiltrating astrocytomas would have loss of ATRX. ATRX IHC for 7 of the 28 cases demonstrated ambiguous staining with variability of labeling across tumor cells and internal control non-neoplastic cells, and were considered uninterpretable; these cases were excluded from further analysis. Out of the 22 IDH-mutated infiltrating astrocytoma cases with interpretable ATRX IHC, 12 showed evidence for loss of ATRX expression in neoplastic cells. Thus, if one were to predict the presence of an ATRX alteration on the basis of Oncomine-detected IDH/TP53 double mutation, this would correlate with immunohistochemically detected ATRX loss of expression in only 55% of cases (Table 3) by our laboratory. Interestingly, when compared to an orthogonal NGS panel (Foundation Medicine) that assessed for ATRX mutation, all 12 of the IDH1/TP53 double mutant cases for which Foundation sequencing was available indeed demonstrated an ATRX mutation. Nine of these alterations were considered known pathogenic alterations and 3 were considered variants of unknown significance (Table 3).

**Table 3:** ATRX status prediction using presence and absence of TP53 and IDH mutations compared to ARTX immunohistochemistry and the FoundationOne targeted next-generation sequencing panel

Infiltrating glioma subgroup	Prediction compared to IHC	Prediction compared to foundation medicine
Oligodendroglioma	13/13 (100%)	3/3 (100%)
IDH-mutant Infiltrating Astrocytoma	12/22 (55%)	9/12–12/12 (75–100%)
IDH-wild type Infiltrating Astrocytoma, presenting in adults	102/102 (100%)	57/63 – 60/63 (91- 95%)

All 14 cases of oligodendroglioma had an IDH mutation detected on Oncomine, but only 2 had *TP53* mutations detected. Given the low incidence of ATRX mutations in oligodendrogliomas, none of these cases were predicted to have loss of ATRX on IHC or Foundation Medicine. IHC for ATRX was available for 13 of the oligodendroglioma cases and all showed preservation of ATRX while only 3 cases (including the one for which IHC was not available) had Foundation Medicine results, none of which showed mutations in ATRX. Therefore, prediction accuracy for oligodendroglioma based on diagnosis and alteration status for IDH status and *TP53* as determined by Oncomine was 100% compared to both IHC and Foundation Medicine results (Table 3).

Of the 112 IDH-wild type infiltrating astrocytoma cases arising in adults, 38 cases did have *TP53* alterations detected by Oncomine. Given the low incidence of ATRX mutations in IDH-wildtype diffuse astrocytomas arising in adults, it was predicted that none of these cases would have ATRX loss by IHC or Foundation Medicine. ATRX IHC was available for 105 of the cases, 3 of which were inconclusive and therefore excluded from further analysis. The 102 cases for which ATRX IHC was available and interpretable, all 102 demonstrated ATRX preservation making the predicted ATRX status relative to ATRX IHC 100% (Table 2). Foundation Medicine results were available for 63 of the 112 cases through which 6 cases were found to have ATRX alterations, 3 were known pathogenic alterations and 3 were classified as VUS. Therefore, the predicted ATRX status was concordant with sequencing in 57/63 (90.5%) cases as compared to Foundation Medicine results (Table 3). Importantly, three of the cases that were discordant between an ATRX prediction metric based upon IDH-wildtype status as determined by Oncomine in adults, and Foundation sequencing, which directly assesses *ATRX* mutations, included 3 cases of pediatric-type hemispheric glioma, H3G34-mutant, all of which presented in young adults in their third decade of life. Of the two cases for which ATRX IHC was performed among these three, both did *not* show loss of ATRX by IHC. As discussed below, while the vast majority of IDH-wildtype gliomas in adults do not have ATRX mutations, evidence of ATRX loss, either by IHC or by sequencing panels that assess this gene should prompt consideration of pediatric-type diffuse gliomas, including H3G34-mutant tumors, and other entities including ‘high grade astrocytoma with piloid features’ (HGAP) [7].

In total, when compared to other targeted-NGS panels that include sequencing of ATRX, the accuracy of ARTX status prediction based on the presence or absence of IDH/TP53 mutations alone is 72/78 (92.3%), indicating the redundancy of this data point in the vast majority of adult cases. Relative to IHC, the presence of IDH1/TP53 double mutation correlated with ATRX loss of expression in 127/137 (93%). The independent clinical utility of assessing for ATRX status by immunohistochemistry in adults is unclear, especially considering that sequencing and/or methylation profiling would typically be required to confirm less common tumor diagnoses in the IDH-wt setting that may harbor ARTX alterations. Moreover, it is unclear the extent to which ATRX expression as measured by IHC at the protein level may be reduced independently from DNA-detectable sequencing alterations of the ATRX gene itself, or on the other hand if loss-of-function mutations may occur even when antigenicity relative to commonly used antibodies in clinical practice is preserved in the translated product.

### Detection of chromosome and gene copy number alterations using oncomine

#### EGFR amplification detection concordance by FISH and oncomine

EGFR amplification was detected by FISH in 39/151 cases for which this assay was performed (25.8%). Oncomine did not detect EGFR amplification in any of the cases with negative FISH results. Of those cases that were called positive for amplification of EGFR by FISH, 35/39 (89.7%) also demonstrated EGFR amplification by Oncomine. The 4 discrepant cases were IDH-wildtype infiltrating astrocytomas which by FISH all had average EGFR signals per nucleus > 4 (range 4.48–6.62) and ratio of EGFR signals/CEP7 signals (a centromeric probe) > 2 (range 2.1 -2.92). Thus, in these ‘discrepant’ cases, tumor cells harbored a relatively low degree of amplification relative to classic cases of EGFR-amplified IDH-wildtype astrocytoma that often harbor 10's to 100's of copies of the gene, often episomally. Interestingly, the copy number of EGFR as inferred by Oncomine analysis for these cases ranged from 2.74 to 4.6 with the copy number ratio of EGFR relative to the average copy number for the remaining genes sequenced by Oncomine on chromosome 7 was between 1.35 and 1.02, more indicative of the increased EGFR copy number being a result of broad chromosomal 7 gain, also a common feature of IDH-wildtype astrocytoma. The biological and prognostic significance of copy number gains and low-level amplification versus high level amplification, and the exact definitional thresholds that should be used as diagnostic criteria (i.e. for gain versus amplification), are not well-defined in the literature and require future studies to further refine. Guidelines published in the cIMPACT-NOW update 3 states that EGFR amplification qualifying for Grade 4 designation of IDH-wildtype astrocytoma in the absence of high-grade histologic features should only be called in the presence of “high-level copy number gains” as established by “clinically validated assays” [9].

#### 1p/19q

**Fig. 2:**
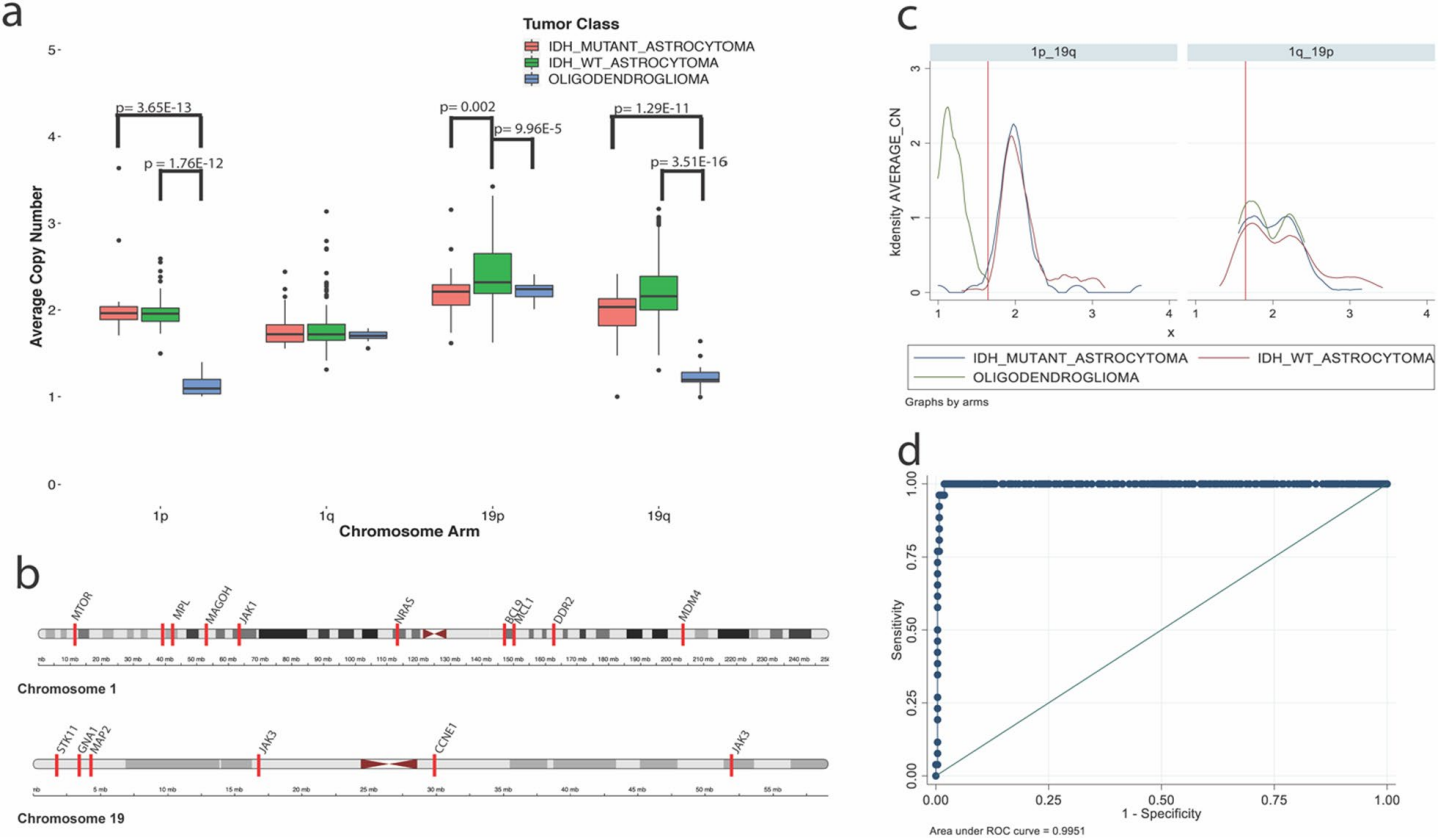
Use of average copy number of genes sequenced by the Oncomine panel located on chromosomes 1p, 1q, 19p, 19q in order to detect 1p/19q co-deletion. **A** Average copy number for genes sequenced on chromosomes 1p, 1q, 19p, and 19q for IDH-mutant infiltrating astrocytoma (IA_IDH_MUTANT), IDH- wild type infiltrating astrocytoma in adults (IA_IDH_WT), and oligodendroglioma (OLIGO). **B** Map of chromosomes 1 and 19 showing distribution of genes sequenced by the Oncomine panel. **C** Average copy number for genes on 1p/19q (left panel) and 1q/19p (right panel) for each subgroup of infiltrating glioma where the red line represents suggested cut-off value of 1.65 for average copy number of genes sequenced on 1p and 19q to detected co-deletion. **D** ROC curve for determination of cut-off value with highest sensitivity (100%) and specificity (98%)

The average copy number for genes tested by the Oncomine panel located on chromosome 1p (MTOR, MYCL, MPL, MAGOH, JAK1, and NRAS) was significantly lower in cases of oligodendroglioma (average CN = 1.15 ± 0.14) compared to IDH-mutant infiltrating astrocytoma (average CN = 2.04 ± 0.37; *p* = 3.65 × 10^–13^) and IDH-wild type infiltrating astrocytoma (average CN = 1.97 ± 0.16; *p* = 1.76 × 10^–12^) but no significant difference was detected between IDH-mutant and IDH-wildtype infiltrating astrocytoma (*p* > 0.05; Fig. 2a). There was no difference in the average copy number for genes tested on chromosome 1q (BCL9, MCL1, DDR2, and MDM4) between the infiltrating glioma subgroups (*p* = 0.44; Fig. 2a).

Unexpectedly, a significant increase in the average copy number of genes tested by Oncomine located on 19p (STK11, GNA11, MAP2K2, and JAK3) was detected in IDH-wildtype infiltrating astrocytoma (average CN = 2.44 ± 0.39) compared to IDH-mutant infiltrating astrocytoma (average CN = 2.22 ± 0.29; *p* = 0.002; Fig. 2a) and oligodendroglioma (average CN = 2.22 ± 0.13; *p* = 9.96 × 10^–5^; Fig. 2a). A significant decrease in average copy number for genes tested on 19q (CCNE1 and PPP2R1A) was noted in oligodendroglioma (average CN = 1.23 ± 0.18) compared to IDH-mutant infiltrating astrocytoma (average CN = 1.96 ± 0.29; *p* = 1.29 × 10^–11^; Fig. 2a) and IDH-wild type infiltrating astrocytoma (average CN = 2.24 ± 0.38; *p* = 3.51 × 10^–16^; Fig. 2a). Interestingly a significant increase in average copy number of 19q was also seen in IDH-wildtype compared to IDH-mutant infiltrating astrocytoma (*p* = 0.0001; Fig. 2a).

In order to determine a cut-off value for average copy number that would be indicative of 1p/19q co-deletion, a receiver operating characteristic (ROC) curve was created by combining the average copy number for all genes sequenced across chromosome 1p and 19q. A cut-off value of 1.65 for average copy number resulted in a sensitivity of 100% and specificity of 98% for detection of 1p/19q co-deletion relative to FISH (Fig. 2c, d). When looking at the average copy number for genes sequenced on chromosome 1p and 19q for each case, there is a distinct difference in the distribution of average copy number for cases of oligodendroglioma versus IDH-mutant and –wild type infiltrating astrocytoma that corresponds to the cut-off at a value of 1.65 (Fig. 2c, d).

#### CDKN2A copy number alterations

**Fig. 3:**
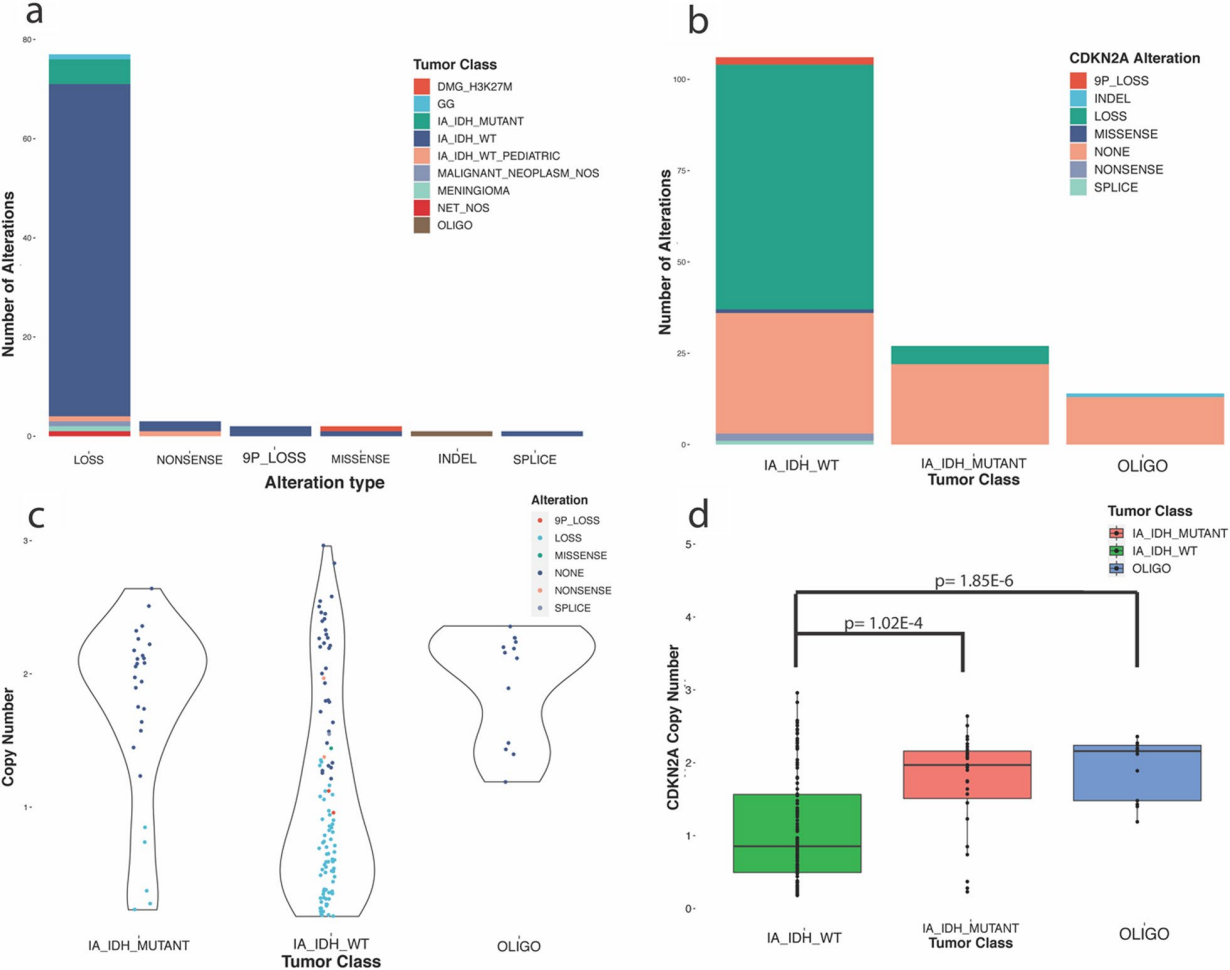
CDKN2A alterations detected on the Oncomine targeted NGS panel. **A** Distribution tumor classes in which each type of alterations detected in CDKN2A was found (DMG_H3K27M = Diffuse midline glioma, H3K27M mutant; GG = Ganglioglioma; IA_IDH_MUTANT = IDH-mutant infiltrating astrocytoma; IA_IDH_WT = IDH-wildtype infiltrating astrocytoma in adult patients; IA_IDH_WT_PEDIATRIC = Infiltrating astrocytomas in pediatric patients, IDH-wildtype; MALIGNANT_NEOPLASM_NOS = malignant neoplasm, not otherwise specified; MENINGIOMA = meningioma; NET_NOS = Neuroepithelial tumor, not otherwise specified; OLIGO = Oligodendroglioma). **B** Types of CDKN2A alterations found in each subgroup of infiltrating glioma. **C** Spread of copy number of CDKN2A by tumor class where each point represents a case which is colored by the CDKN2A alteration class called by the Oncomine panel. **D** distribution of copy number of CDKN2A for subgroups of infiltrating glioma

The Oncomine panel detected 86 alterations in the *CDKN2A* gene over all cases. The majority of these alterations were isolated copy number losses in *CDKN2A* (77/86; 89.5%) along with 2 cases where evidence for a putative deletion more broadly over the 9p chromosomal arm was detected (2/86; 2.3%). Three cases had a nonsense variant detected (3.5%) while 2 had a missense mutation detected (2.3%). Additionally, a frame shift deletion was detected in one case and a splice site variation in another (1.2% each). The distribution of tumor classes for each alteration detected in *CDKN2A* by the Oncomine panel can be seen in Fig. 3a and b.

Out of the 219 cases that the copy number of CDKN2A was recorded, loss of CDKN2A was detected in 77 (35.2%). Of these, the vast majority were IDH-wildtype infiltrating astrocytoma (67/77; 87.0%) with IDH mutant infiltrating astrocytoma being the second most common tumor type for which CDKN2A loss was detected by targeted NGS (5/77; 6.5%). The remaining 5 cases in which loss of CDKN2A was detected comprised one ganglioglioma, one pediatric-type IDH-wildtype infiltrating astrocytoma, one meningioma, one neuroepithelial tumor not otherwise specified (NOS), and one ‘malignant neoplasm, NOS’ (Fig. 3a, b).

Of 106 cases of IDH-wildtype infiltrating astrocytoma with copy number data for CDKN2A, loss was seen in 67 cases (63.2%). Only 5 of 27 (19%) cases of IDH-mutant cases had loss of CDKN2A detected and 0 of 13 cases of oligodendroglioma had a loss in CDKN2A, although one case demonstrated a frame-shift deletion alteration (Fig. 3b, c).

The average copy number for CDKN2A was 1.09 ± 0.75 in IDH-wildtype infiltrating astrocytoma, 1.73 ± 0.68 in IDH-mutant infiltrating astrocytoma, and 1.94 ± 0.41 in oligodendroglioma. When comparing these values using paired t-tests, the average copy number for IDH-wildtype infiltrating astrocytoma was lower than in IDH-mutant infiltrating astrocytoma (*p* = 1.02 × 10^–4^) and oligodendroglioma (*p* = 1.85 × 10^–6^). No significant difference was found in the copy numbers between IDH-mutant infiltrating astrocytoma and oligodendroglioma overall (*p* = 0.24) (Fig. 3d).

### Added-value of RNA-seq component of oncomine

**Fig. 4:**
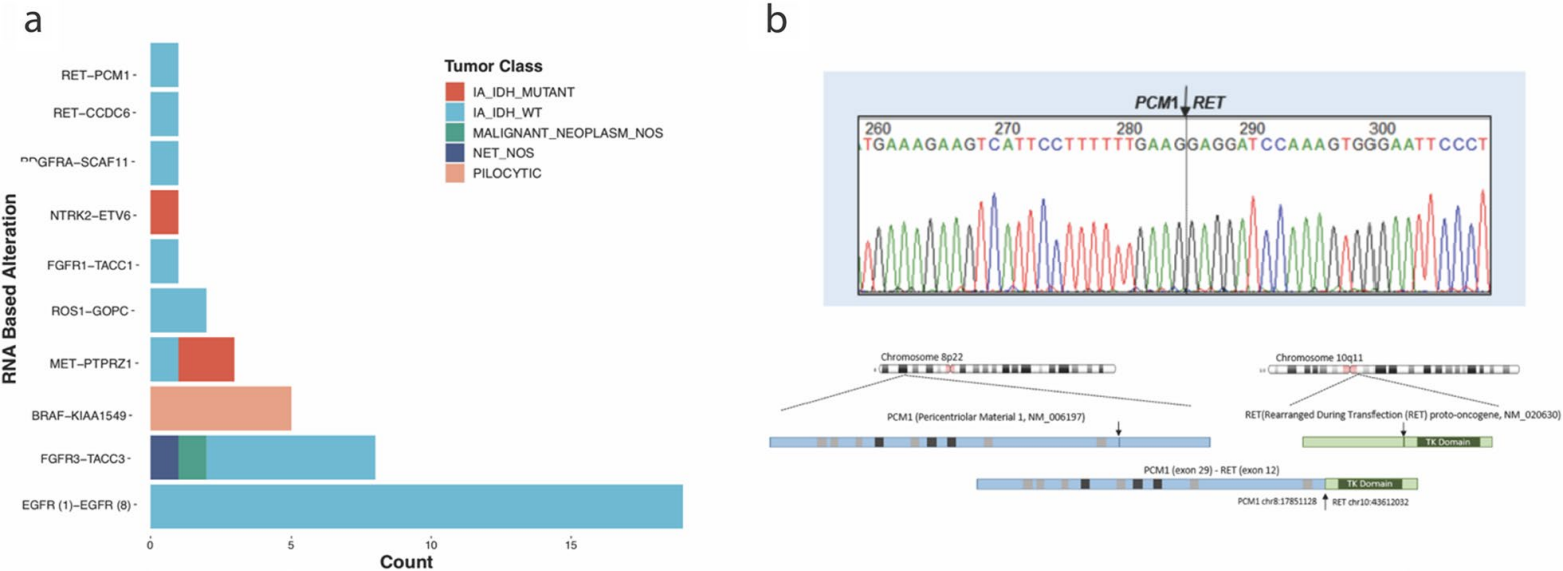
RNA based alterations detected in the RNA-Seq portion of the Oncomine targeted next-generation sequencing panel. **A** All detected fusions with column coloration by diagnoses for which each alteration as found (IA_IDH_MUTANT = IDH-mutant infiltrating astrocytoma; IA_IDH_WT = IDH-wildtype infiltrating astrocytoma in adults; MALIGNANT_NEOPLASM_NOS = malignant neoplasm, not otherwise specified; NET_NOS = neuroepithelial tumor, not otherwise specified; PILOCYTIC = pilocytic astrocytoma). **B** Sanger sequencing confirming RET-PCM1 fusion and chromosomal structure of the fusion

The RNA-Seq portion of the Oncomine panel detected 42 RNA-based alterations from the broader cohort of 233 CNS tumor cases, including 157 infiltrating gliomas (Fig. 4a). The RNA-Seq portion of the Oncomine panel failed in 21/233 cases (9.01%) due to either poor RNA quality or low quantity. All detected fusions were confirmed by orthogonal RT-PCR analysis using site-specific primers (data not shown). The alterations detected by the panel included EGFRvIII, (EGFR transcripts with a deletion of exons 2–7) that is characteristic of a subset of IDH-wildtype GBM, and indeed all 19 cases with this transcript detected were IDH-wildtype GBM (Fig. 4a)*.* Interestingly, several additional fusion transcripts were detected by the Oncomine panel, a generic solid tumor panel that was not designed specifically for tumors of the CNS. Some of the detected fusions have been previously reported in infiltrating gliomas, including MET-PTPRZ1, which was detected in 3 cases (2 IDH-mutant and 1 IDH-wildtype case), FGFR1-TACC1 (1 IDH-wildtype case), and FGFR3-TACC3 in 8 cases (6 IDH-wildtype infiltrating astrocytomas, 1 neuroendocrine tumor NOS, and one malignant neoplasm, NOS) (Fig. 4a). Additional less frequently reported alterations were detected in infiltrating gliomas including an NTRK2-ETV6 fusion (1 IDH-mutant astrocytoma) and ROS1-GOPC fusions (2 in IDH-wildtype infiltrating astrocytomas).

Several of the fusions detected that, to our knowledge, have not been previously reported in CNS tumors included PDGFRA-SCAF11 as well as 2 RET fusions, RET-CCDC6 and RET-PCM1, all of which occurred in cases of IDH-wildtype infiltrating astrocytoma (Fig. 4a, b). Of particular interest is that in both cases with RET fusions, these patients had additional germline alterations in BRCA genes: BRCA2 S1982fs in the RET-PCM1 fused case and BRCA1 splice site 442-1G > T in the RET-CCDC6 fused case. Notably, the RET-PCM1 fusion was detected in a surgically resected recurrent tumor that had followed prior surgery, radiation therapy, and temozolomide treatment. The patient subsequently was treated with one cycle of CCNU through which her tumor progressed. The option of enrolling in a clinical trial for targeted treatment of RET fusion was declined by the patient. The elderly patient harboring the RET-CCDC6 fusion also did not receive additional treatment based on the sequencing results given several clinical factors that led to a decision to forgo additional treatment.

In 5/11 cases of pilocytic astrocytoma a BRAF-KIAA1549 fusion was detected by the panel. One case of pilocytic astrocytoma had a BRAF-KIAA1549 fusion detected by Foundation Medicine in addition to FISH demonstrating tandem duplication of the BRAF locus, but not by the Oncomine panel. This discrepancy with Foundation medicine may be a result of differences in primer design for amplifying transcript reads pertaining only to certain predetermined fusion sites. Of the remaining cases of pilocytic astrocytoma for which BRAF-KIAA1549 fusion was not found, additional drivers such as FGFR4 missense and NF1 truncating mutations were found (1 case each). In only one case of pilocytic astrocytoma were no variants detected by Oncomine.

## Discussion

Integrating molecular data with histopathologic examination of brain tumors has resulted in refined diagnostic criteria and improved prognostication of CNS tumors including infiltrating glioma, directly influencing clinical management. In the clinical laboratory, while newer diagnostic modalities are brought online, older methodologies are often retained and performed in tandem, with the potential to duplicate effort and waste resources. In fact, the cost of standard work-up for infiltrating gliomas using IHC, FISH, and Oncomine at our institution was 2.24 × higher than Oncomine alone (Table 4). Streamlining the standard diagnostic work-up has the potential to decrease tissue waste, decrease cost, and prevent redundant or even confounding results between two separate analyses.

**Table 4:** Cost of typical diagnostic work-up for infiltrating glioma cases including IHC for GFAP, IDH1-R132H, p53, ATRX, and Ki-67; FISH (for 1p/19q assessment, EGFR assessment, and CDK2NA assessment); and cost of running and interpretation of the Oncomine targeted NGS panel, as compared to the Oncomine panel-only, as well as IHC and FISH studies only

Diagnostic test	Tech time and reagents	Professional component–medicare reimbursement	Total cost
IHC (5 IHC)	$26 × 5 = $130	(IHC code 342 × 1 at $34) + (IHC code 341 × 3 at $28) + (IHC code 360 × 1 at $41) = $159	$289
FISH (1p/1q, 19p/19q, *EGFR/centromeric*, *CDKN2A/centromeric*)	$127 × 4 = $508	FISH code 377 × 4 at $63 = $252	$760
NGS (Oncomine v2)	$700	$48	$748

Total cost of diagnostic work-up			Total cost
Standard (IHC, FISH, and NGS)	$1338	$459	$1797
NGS alone	$700	$48	$748
IHC and FISH	$638	$411	$1049

The costs listed reflect an estimate of technician time and reagents, plus the Medicare reimbursement rate of the professional component as a proxy for a standardized interpretation portion of the cost. We recognize that these values can vary widely across institutions. NGS costs are calculated on a per-case cost with the assumption that full batches are run

Our results demonstrate that a general solid tumor targeted NGS panel is more sensitive than IHC for detection of IDH1/2 alterations and is a more robust assay for detecting and interpreting TP53 mutations. Moreover, we have shown that in > 90% of cases, a combination of IDH, TP53, and codeletion status predicts the status of ATRX alterations relative to direct sequencing assessment, and that for the class of IDH-mutated astrocytomas there is no correlation between staining pattern and sequencing prediction. We also show that sequencing-derived copy number status of the 1p/19q chromosomal arms and EGFR amplification status performs as well as standard FISH, and has the added benefit of detecting diagnostically critical additional copy number alterations, including CDKN2A deletion, with regularity. We also demonstrate the additional benefit of including an RNA sequencing component in a targeted NGS panel by detecting potentially targetable fusion transcripts even using a panel not specifically designed for CNS tumors.

In particular, while immunohistochemical staining has repeatedly been shown to be reliable for detecting the IDH1 R132H variant, with reported sensitivity around 94%, specificity of up to 100%, and concordance of 98% with sequencing [11, 12, 15, 25, 26, 37], 10% of IDH mutations in infiltrating gliomas are non-R132H variants and will therefore be missed by IHC alone. These findings were recapitulated in our data where 9.3% of cases with IDH1/2 mutations were missed by IHC, including IDH1 R132S, IDH1 R132G, and IDH2 R172H. We argue that with this rate of false negatives, particularly in younger age groups, a case can be made for performing sequencing alone since all negatives would need to be sequenced, and moreover the tumor would likely undergo sequencing in institutions that perform NGS panels regardless of the status of the stain.

TP53 mutational status can help to differentiate between IDH-mutant infiltrating astrocytoma and oligodendroglioma in cases where the presence of 1p/19q co-deletion is either not available or unclear in the setting of complex copy number alterations. Immunohistochemistry is often used for the evaluation of TP53 mutation, but interpretation of p53 IHC staining can be challenging and misleading [33, 49]. Missense mutation in TP53 results in slower degradation of the p53 protein and therefore sustained nuclear build-up resulting in increased labeling while lack of staining may correlate with a truncating mutation resulting in no production of the p53 epitope recognized by the laboratory antibody [33, 49]. The expression of TP53 is tightly regulated and normally expressed in various levels depending on tissue type, therefore determining whether p53 IHC staining is more or less than expected compared to non-lesional tissue is often complicated [33, 49]. This difficulty was similarly demonstrated in our own data, where the accuracy for predicting wild-type TP53 status based on IHC was only 82.4% relative to sequencing out of all cases evaluated. We advocate for the increased use of targeted sequencing for determining the presence of TP53 mutation given that IHC for p53 provides little additional value.

While FISH is a well-established laboratory technique used for the evaluation of copy number variants and chromosomal structural alterations, inherent pitfalls in the methodology can affect its utility. In particular, the probe used in FISH analysis typically only targets a specific locus on a given chromosomal arm, and focal deletions may result in “false positive” results, for example with respect to the canonical *whole arm loss* that is used to define oligodendrogliomas and that results from an unbalanced translocation of chromosomes 1 and 19. Various groups have looked at other means of detecting 1p/19q co-deletion utilizing results from targeted NGS, including detecting loss of heterozygosity by examining single nucleotide polymorphisms and use of deep learning convolutional neural networks to predict 1p/19q co-deletion from results of targeted NGS [18, 21, 34]. In this study, we found that averaging the estimated copy number of genes sequenced by the Oncomine panel on chromosomes 1p, 1q, 19p, and 19q robustly detect 1p/19q co-deletion with a sensitivity of 100% and specificity of 98% using a cut-off average copy number of 1.65. The viability of replacing FISH with targeted-NGS would have logistical and practical benefits in the diagnostic work-up of infiltrating gliomas.

Oncomine features an RNA sequencing component which can identify important fusions or other RNA-based alterations. Along with the identification of common RNA-based alterations associated with certain CNS neoplasms, such as EGFRvIII in IDH-wild type glioblastoma and BRAF-KIAA1549 fusions in pilocytic astrocytoma, we also detected a number of rare fusions in our cohort for which targeted treatments are potentially available [2, 6, 16]. This includes GOPC-ROS fusions for which ROS inhibitors such as crizotinib and lorlatinib are available, RET fusions with CCDC6 and PCM1 which may be amendable to cabozanitinib and vandetanib therapies, ETV-NTRK fusions for which larotrecitinib and entrectinib have been used, and MET-PTPRZ1 fusions which have been implicated in IDH-mutant high grade astrocytomas and for which MET-specific kinase inhibitors are under investigation [13, 17, 19, 27, 30, 40, 43]. With the exception of crizotinib, these targeted therapies have blood–brain barrier penetrance [5, 20, 23, 35, 42, 44]. Despite the fact that the Oncomine panel is limited to the detection of fusions for which both fusion partners are known and that it was not designed specifically for CNS tumors, a number of important and potentially targetable RNA-based alterations were detected, advocating for the expanded use of RNA sequencing as part of the standard work-up of CNS neoplasms. The value of RNA sequencing could be further enhanced by use of platforms such as Archer FusionPlex, which does not require knowledge of fusion partners, allowing for the detection of novel fusions, and/or by large panels that are designed to assess the ever expanding list of fusions that are recurrently detected in tumors of the CNS [24].

While the Oncomine v2 panel has demonstrated broad utility in the diagnostic work-up of CNS tumors, this particular panel presents several shortcomings which argue for the validation of a more comprehensive targeted panel if possible. For example, the Oncomine v2 panel lacks evaluation of the TERT promoter, which is diagnostically critical for glioblastoma and CNS WHO grade 3 meningiomas, and it is recurrently altered other tumors, including oligodendroglioma. At our own institution, in the past we have supplemented the Oncomine panel with stand-alone sequencing of the *TERT* promoter. More, recently we have validated an expanded targeted panel, the TruSight Oncology 500 Assay (TSO500) that includes coverage of *TERT* as well as other relevant genes not covered by Oncomine v2, including *ATRX* and *H3F3A.*

As different targeted panels employ slightly different methodologies, it is worth considering the advantages and disadvantages of amplicon-based methods versus hybridization capture-based methods. The Oncomine panel uses amplicon-based sequencing, a technology that is generally faster and cheaper, and better-suited to smaller panels where there is reduced risk of primer interference. Hybridization capture-based techniques are generally more robust, but they are more labor-intensive with longer protocols for library preparation, sequencing, and analysis, all of which can negatively affect turnaround time.

A major benefit of hybridization capture techniques over the amplicon-based strategies concerns the RNA-based component of the assay and fusion detection. Because amplicon-based methods are dependent on specific primers, where one primer hybridizes to the putative driver gene, and the other to its partner, so that the resulting amplicon (fusion contig) includes the breakpoint, both fusion partners must be known. In contrast, both hybridization capture and anchored multiplex PCR techniques are fusion partner-agnostic. For hybridization capture, the fusion is pulled down using probes that are complementary to the gene of interest, and in so doing, any fusion partner, including novel fusion partners, will be pulled down as well. In anchored multiplex PCR, RNA adaptors are added to the ends of cDNA that hybridize a universal primer. PCR is then performed using the universal primer and a driver gene-specific primer.

While turnaround time for IHC is of course significantly faster than NGS, typically on the order of 5 to 20 days depending on the complexity and batching requirements of the NGS assays employed, in our own practice IHC is very rarely relied upon in and of itself for diagnostic purposes. For example, given that 10% of IDH mutations are not detected by the IDH1 R132H stain, the final diagnoses for immuno-negative cases (particularly in younger populations) would be contingent on waiting for the NGS data. A diagnosis of an H3G34-mutant glioma or HGAP would not be made on the basis of ATRX IHC even in cases where those diagnoses are suspected, and sequencing or methylation profiling would be necessary, respectively. We do agree that in certain circumstances, such as with a positive H3K27M stain or a positive triple-mutated IDH1-mutant/ATRX-lost/p53-mutant tumor, the immunoprofile can very quickly yield a definitive diagnosis; however, it is not clear that there is a clinically significant benefit to having this diagnosis in 2–3 days (IHC) versus 7–10 days (expedited NGS) versus 10–21 days (routine TAT for NGS at our institution) to merit routine use. Our institution’s current turn-around time is 10–14 days for the Oncomine panel and 21 days for TSO500. For selected cases, upon clinical request (due to clinical trial eligibility demands for example) we have the ability to expedite TAT at the expense of running an incomplete batch, though this can be costly to execute on a routine basis.

## Conclusions

The ability to more precisely classify and accurately prognosticate infiltrating gliomas has improved significantly over the past decade due to the discovery of molecular alterations that now define certain diagnostic entities and influence their biologic behavior. The detection and reporting of alterations such as IDH1/2 mutation, 1p/19q co-deletion, EGFR amplification, TERT-promoter mutation, and CDKN2A homozygous loss in the diagnosis of infiltrating glioma has become standard practice in clinical neuropathology. The improvement in our understanding and diagnostic ability is in part due to the advancement of molecular diagnostic techniques, such as targeted NGS, allowing for the simultaneous sequencing of multiple genes implicated in oncogenesis. Despite such advancements, pathologists still rely on older methodologies such as IHC and FISH, which are not significantly less expensive in aggregate, are often less precise, and are operator dependent, occasionally leading to conflicting results.

We sought to answer whether increased reliance on targeted NGS panels can provide as much information as the use of multiple modalities, such as IHC and FISH, and detect additional molecular alterations of diagnostic and therapeutic importance. The work presented here shows that targeted NGS was superior to IHC for the detection of mutations in IDH1/2 and TP53. We also show that utilizing copy number data obtained by targeted NGS detects EGFR amplification as well as 1p/19q co-deletion similarly to FISH and can detect additional copy number alterations in prognostically important genes, such as CDKN2A. Along with demonstrating that targeted NGS panels can more reliably detect molecular alterations commonly assessed by IHC and FISH, we also show that the addition of an RNA sequencing component in targeted NGS panels detects fusion transcripts for which there may be targeted therapeutic approaches available. With the iterative improvement and adoption of NGS panels to include a broader gene set on the order of 500 genes, and with fusion-partner agnostic transcript assessment modalities, the cost effectiveness and diagnostic efficiency of panels will only improve and will continue to render redundant, if not obsolete, more conventional diagnostic modalities.

## Supplementary Information

**Additional file 1:** **Table S1.** A list of antibody clones, dilutions, and antigen retrieval methods used for IDH1, p53, and ATRX. **Table S2:**  A list of genes covered by the Oncomine Comprensive Panel v2.

**Additional file 2:** **Figure S1.** Immunohistochemical staining patterns for IDH1 R132H (top row), ATRX (middle row), and p53 (bottom row). IHC for IDH1 R132H was classified as either positive or negative. The staining pattern for ATRX was designated as either preserved if nuclear staining was present, lost if there was no nuclear staining, or inconclusive if staining was present in some tumor cells but absent in others. Staining for p53 was separated into one of four categories: 0 if there was absent staining for p53 indicative of a truncating mutation, 1 if the pattern of staining was as expected for central nervous system tissue and therefore not concerning for an underlying mutation, 2 if the stain was inconclusive or concerning for a subclonal mutation, or 3 if there was strong staining in a large number of tumor cells concerning for the presence of an underlying missense mutation.

## Data Availability

The datasets generated and/or analyzed during the current study were generated from pathology specimens at New York Presbyterian Hospital/Weill Cornell Medicine and are not publicly available but are available from the corresponding author on reasonable request and upon completion of any necessary interinstitutional Materials Transfer Agreement.

## Abbreviations

WHO: World health organization
IDH: Isocitrate dehydrogenase
FISH: Fluorescence in situ hybridization
NGS: Next-generation sequencing
IHC: Immunohistochemistry
ROC: Receiver operator curve
