# Supplementary material for: Towards a single-assay approach: a combined DNA/RNA sequencing panel eliminates diagnostic redundancy and detects clinically-relevant fusions in neuropathology
Source: Acta Neuropathol Commun. 2022 Nov 17;10:167. doi: 10.1186/s40478-022-01466-w (PMC9670552; doi:10.1186/s40478-022-01466-w)
Supplement: Supplementary file 1 — Additional file 1: Table S1. A list of antibody clones, dilutions, and antigen retrieval methods used for IDH1, p53, and ATRX. Table S2: A list of genes covered by the Oncomine Comprensive Panel v2. [file 40478_2022_1466_MOESM1_ESM.docx]

**Supplementary Materials:**

Supplemental Table 1: Clone, dilation, and antigen retrieval for IDH1 R132H, p53, and ATRX immunohistochemical stains.

| **Antibody** | **Clone** | **Manufacturer** | **Dilution** | **Antigen Retrieval** |
| --- | --- | --- | --- | --- |
| IDH1 | H09 | Dianova | 1:25 | H2(20) |
| p53 | 180-1 | Cell Signaling | 1:400 | H2(20) |
| ATRX | polyclonal | Sigma | 1:100 | H1(30) |

Supplementary Table 2: List of genes covered in Oncomine Comprehensive Panel v2.

| **Hot Spot Genes** | | | | |
| --- | --- | --- | --- | --- |
| ABL1 | ERBB2 | HRAS | MAPK1 | PTPN11 |
| AKT1 | ERRB3 | IDH1 | MAX | RAC1 |
| ALK | ERRB4 | IDH2 | MED12 | RAF1 |
| AR | ESR1 | IFITM1 | MET | RET |
| ARAF | EZH2 | IFITM3 | MLH1 | RHEB |
| BRAF | FGFR1 | JAK1 | MPL | RHOA |
| BTK | FGFR2 | JAK2 | MTOR | SF3B1 |
| CBL | FGFR3 | JAK3 | MYD88 | SMO |
| CDK4 | FLT3 | KDR | NFE2L2 | SPOP |
| CHEK2 | FOXL2 | KIT | NPM1 | SRC |
| CSF1R | GATA2 | KNSTRN | NRAS | STAT3 |
| CTNNB1 | GNA11 | KRAS | PAX5 | U2AF1 |
| DDR2 | GNAQ | MAGOH | PDGFRA | XPO1 |
| DNMT3A | GNAS | MAP2K1 | PIK3CA |  |
| EGFR | HNF1A | MAP2K2 | PPP2R1A |  |
| **Full Length Genes** | | | | |
| APC | CDKN2A | NOTCH1 | SMARCB1 | VHL |
| ATM | FBXW7 | PIK3R1 | STK11 | WT1 |
| BAP1 | GATA3 | PTCH1 | TET2 |  |
| BRCA1 | MSH2 | PTEN | TP53 |  |
| BRCA2 | NF1 | RB1 | TSC1 |  |
| CDH1 | NF2 | SMAD4 | TSC2 |  |
| **Copy Number Variants** | | | | |
| ACVRL1 | CCNE1 | FGFR2 | MDM2 | PDGFRA |
| AKT1 | CD274 | FGFR3 | MDM4 | PIK3CA |
| APEX1 | CD44 | FGFR4 | MET | PNP |
| AR | CDK4 | FLT3 | MYC | PPARG |
| ATP11B | CDK6 | GAS6 | MYCL | SMARCB1 |
| BCL2L1 | CSNK2A1 | IGF1R | MYCN | SOX2 |
| BCL9 | DCUN1D1 | IL6 | MYO18A | TERT |
| BIRC2 | EGFR | KIT | NKX2-1 | TIAF1 |
| BIRC3 | ERBB2 | KRAS | NKX2-8 | ZNF217 |
| CCND1 | FGFR1 | MCL1 | PDCD1LG2 |  |
| **Fusion Genes Assessed (with only a subset of known, common partners tested)** | | | | |
| ABL1 | ERG | ERBB2 | NTRK1 | RAF1 |
| AKT2 | ETV1 | FGFR1 | NTRK2 | RET |
| ALK | ETV4 | FGFR2 | NTRK3 | ROS1 |
| AXL | ETV5 | FGFR3 | PDGFRA |  |
| BRAF | EGFR | MET | PPARG |  |
