# Supplementary figures and images for: Towards a single-assay approach: a combined DNA/RNA sequencing panel eliminates diagnostic redundancy and detects clinically-relevant fusions in neuropathology
Source: Acta Neuropathol Commun. 2022 Nov 17;10:167. doi: 10.1186/s40478-022-01466-w (PMC9670552; doi:10.1186/s40478-022-01466-w)

IDH1 R132H

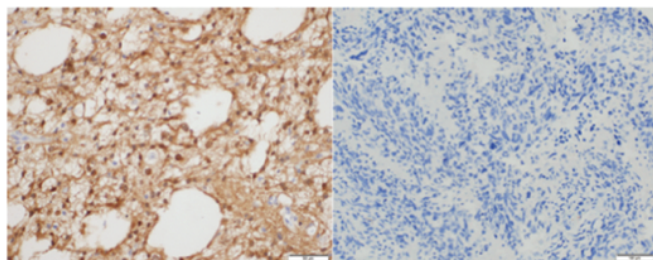

Positive

Negative

ATRX

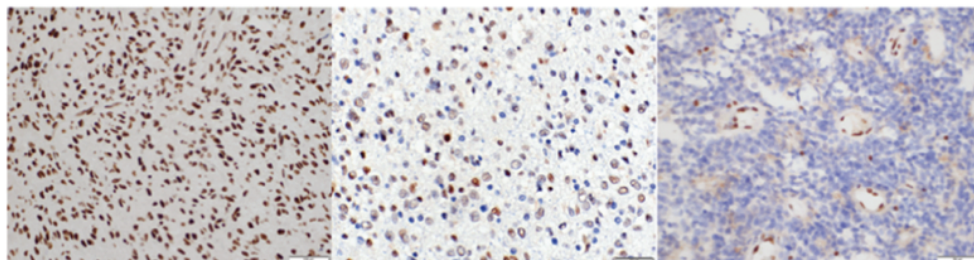

Preserved

Inconclusive

Lost

p53

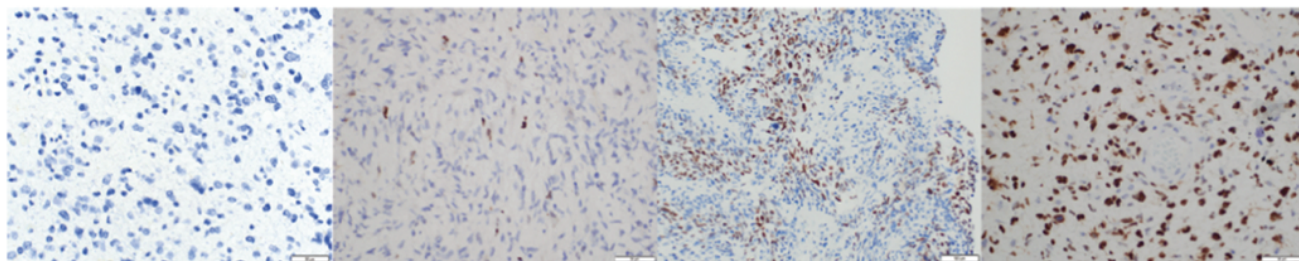

0

1

2

3

Supplement: Supplementary file 2 — Additional file 2: Figure S1. Immunohistochemical staining patterns for IDH1 R132H (top row), ATRX (middle row), and p53 (bottom row). IHC for IDH1 R132H was classified as either positive or negative. The staining pattern for ATRX was designated as either preserved if nuclear staining was present, lost if there was no nuclear staining, or inconclusive if staining was present in some tumor cells but absent in others. Staining for p53 was separated into one of four categories: 0 if there was absent staining for p53 indicative of a truncating mutation, 1 if the pattern of staining was as expected for central nervous system tissue and therefore not concerning for an underlying mutation, 2 if the stain was inconclusive or concerning for a subclonal mutation, or 3 if there was strong staining in a large number of tumor cells concerning for the presence of an underlying missense mutation. [file 40478_2022_1466_MOESM2_ESM.pdf]
